# Platinum-free, graphene based anodes and air cathodes for single chamber microbial fuel cells†
†Electronic supplementary information (ESI) available. See DOI: 10.1039/c7ta06895f


**DOI:** 10.1039/c7ta06895f

**Published:** 2017-11-02

**Authors:** Toby P. Call, Tian Carey, Paolo Bombelli, David J. Lea-Smith, Philippa Hooper, Christopher J. Howe, Felice Torrisi

**Affiliations:** a Department of Biochemistry , University of Cambridge , Hopkins Building, Downing Site, Tennis Court Road , Cambridge , CB2 1QW , UK . Email: ch26@cam.ac.uk ; Fax: +44 (0)1223 333 345 ; Tel: +44 (0)1223 333688; b Cambridge Graphene Centre , Department of Engineering , University of Cambridge , 9 JJ Thomson Avenue , Cambridge , CB3 0FA , UK . Email: ft242@cam.ac.uk ; Fax: +44 (0)1223 748348 ; Tel: +44 (0)1223 332803; c Department of Chemical Engineering and Biotechnology , University of Cambridge , Philippa Fawcett Drive , Cambridge , CB3 0AS , UK

## Abstract

Microbial fuel cells (MFCs) exploit the ability of microorganisms to generate electrical power during metabolism of substrates. However, the low efficiency of extracellular electron transfer from cells to the anode and the use of expensive rare metals as catalysts, such as platinum, limit their application and scalability. In this study we investigate the use of pristine graphene based electrodes at both the anode and the cathode of a MFC for efficient electrical energy production from the metabolically versatile bacterium *Rhodopseudomonas palustris* CGA009. We achieve a volumetric peak power output (*P*_V_) of up to 3.51 ± 0.50 W m^–3^ using graphene based aerogel anodes with a surface area of 8.2 m^2^ g^–1^. We demonstrate that enhanced MFC output arises from the interplay of the improved surface area, enhanced conductivity, and catalytic surface groups of the graphene based electrode. In addition, we show a 500-fold increase in *P*_V_ to 1.3 ± 0.23 W m^–3^ when using a graphene coated stainless steel (SS) air cathode, compared to an uncoated SS cathode, demonstrating the feasibility of a platinum-free, graphene catalysed MFCs. Finally, we show a direct application for microwatt-consuming electronics by connecting several of these coin sized devices in series to power a digital clock.

## Abstract 2

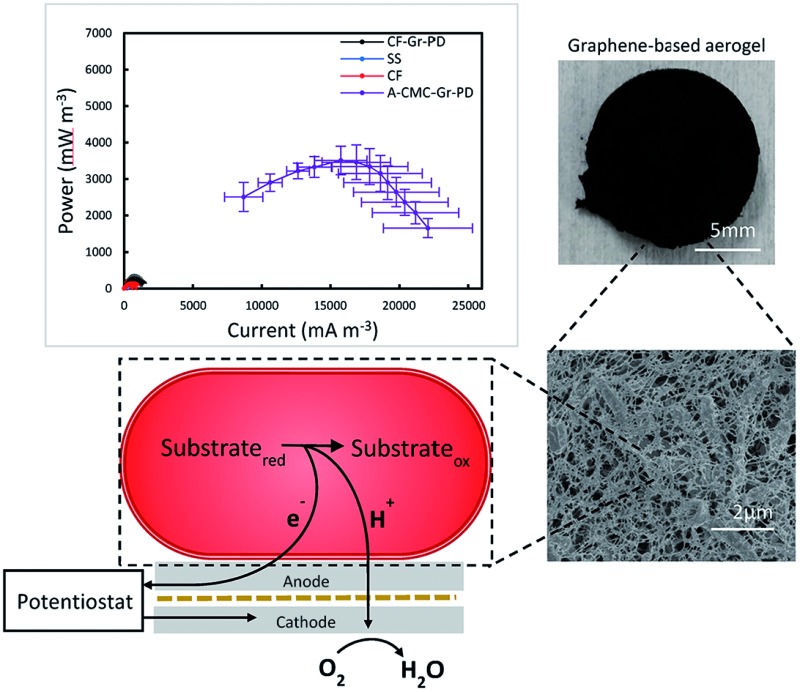
In this work graphene-based aerogel anodes and graphene/stainless steel cathodes have been optimised as platinum-free electrodes in *Rhodopseudomonas palustris* microbial fuel cells, achieving a maximum power output of ∼3.5 W m^–3^.

## Introduction

Global population expansion and economic development result in increasing demand for energy and clean water, leading to a pressing need for innovative renewable energy sources and more efficient and sustainable waste treatment technologies. Microbial fuel cell (MFC) technology may satisfy both requirements by tapping into the significant chemical energy in wastewater, exploiting the electrogenic nature of various microorganisms that oxidize organic substrates and donate electrons to an external electron acceptor. Although examples of carbon-based anodes and platinum (Pt) cathodes exist, there remains major scope for improving the performance of electrodes for MFCs. Enhanced understanding of the parameters determining electrode performance will help in the development of environmentally-friendly, abundant catalytic cathode materials, and highly electron-accepting anode materials. Here we identify key parameters of a range of MFC electrodes and characterise the performance of a set of novel, environmentally-friendly low-cost graphene-based anodes and cathodes.

Power output from a single chamber MFC is primarily limited by the efficiency of extracellular electron transfer (EET) from the cell to the anode,^1^–^4^ mass transport of protons to the cathode,^4^–^6^ and the catalytic efficiency of the oxygen reduction reaction (ORR) at the air cathode.^7^,^8^ Therefore, ideal anode materials for a single chamber MFC should maximize conductivity^1^ and surface area^9^ to facilitate current generation *via* direct extracellular electron transfer (DEET) from anodic biofilms^10^ and efficiently catalyse H_2_O formation and evaporation *via* ORR at the air exposed cathode.^11^

Cost-effective carbon-based materials such as carbon felt,^2^ carbon fibre,^12^ carbon paper,^9^ and graphite^13^ have been used extensively as anode materials due to their chemical stability (*i.e.* their resistance to corrosion in an aqueous environment), surface area (∼0.5 m^2^ g^–1^) and electrical conductivity.^1^ Pt is often incorporated with materials such as carbon paper as an optimal air cathode catalyst for laboratory scale MFCs. However, high costs prohibit scale up using Pt-based catalysts.^14^ Given the need to develop low-cost, environmentally-friendly applications in MFCs there is a growing interest in graphene-based electrodes. Carbon nanotubes^15^ and graphene are at the forefront of research in electronics,^16^ energy^17^ and photonics.^18^ Graphene has a theoretical surface area of 2630 m^2^ g^–1^ (∼5000 times higher than traditional anode materials),^19^ potential for cost-effective mass production,^20^ unique electrical conductivity,^21^ catalytic activity,^22^ and mechanical strength.^23^ These properties, combined with the ease of functionalization^24^ and biocompatibility,^25^ promise to widen the potential range of applications of graphene based MFCs including incorporation in wastewater treatment plants for pathogen reduction,^26^ biological oxygen demand biosensors^27^ and powering implantable medical devices.^28^ Additionally, oxygen and nitrogen-containing functional groups (present in graphene oxide – GO-produced by the modified Hummers method^29^ and chemical vapour deposition^22^) have been reported to impart a catalytic effect to graphene oxide, improving EET efficiency *via* an electron shuttling processes, and potentially providing an alternative and cheaper cathodic catalyst to commonly used Pt^30^,^31^ (cost ∼ £26 g^–1^). MFC anodes using chemically modified GO have been shown to enhance power densities to 2.67 W m^–2^ (∼18-fold) for stainless steel mesh,^32^ to 0.0525 W m^–3^ (∼3-fold) from carbon cloth,^33^ and to 661 W m^–3^ (∼19-fold) for nickel oxide foams.^3^ Recently, an MFC operated with a modified GO-based aerogel anode achieved the highest volumetric power density reported to date,^34^ 750 W m^–3^ (normalised to anode volume). Their low density and high surface area, together with high conductivity, establish aerogels based on GO as high performance MFC anodes.^13^,^35^,^36^ However, GO suffers from defects induced into graphene's basal plane from chemical oxidation, significantly impairing its mechanical and electrical properties. Chemical or thermal reduction to reduced GO (RGO)^31^,^37^ only partially recovers the mechanical and electrical properties of graphene. Pristine graphene with an unaltered basal plane has been grown by chemical vapour deposition (CVD) on a nickel mesh template to create conductive and porous (∼850 m^2^ g^–1^) structures.^38^ However, the mesh template, usually copper or nickel, often requires intensive procedures including an acid etching step for removal (which can create chemical residuals), and gas precursors (*e.g.* methane) for CVD,^39^ substantially increasing costs of electrode fabrication. Pristine graphene flake based aerogels created by freeze gelation of solvent/graphene solutions offer a simple alternative, with superior electrical properties to GO/RGO aerogels.^40^ Despite being labelled as electrochemically inert^41^ and lacking the density of functional groups present on GO/RGO, pristine graphene can catalyse the reduction of oxygen.^42^ Molecular oxygen (i) binds ionically to graphene followed by (ii) endothermic formation of two covalent bonds in an intermediate metastable configuration, energetically favourable (iii) separation of the oxygen atoms to form two epoxy groups on the graphene lattice, and (iv) formation of hydroxyl groups and release of H_2_O.^43^ Additionally, the incorporation of conductive polymers into the backbone scaffold of the aerogel may help bridge the graphene flakes and help to maintain mechanical integrity. Poly(3,4-ethylenedioxythiophene)-poly(styrenesulfonate) (PD) is an interesting MFC electrode material thanks to its high conductivity (∼5 × 10^4^ S m^–1^), and positively charged backbone that may interact electrostatically with negatively charged cells to facilitate cell–anode interactions and biofilm formation.^44^

Pristine graphene can be produced sustainably by liquid phase exfoliation (LPE) or cracking of methane biogas (derived from food waste and other renewable sources) both of which we used to produce low-cost, scalable, and environmentally friendly electrodes for MFCs. In the anodic chamber we use the metabolically diverse, purple non-sulphur α-proteobacterium, *Rhodopseudomonas palustris* (*R. palustris*) CGA009 (ref. 45, 46 and 47). *R. palustris* has been shown to express electrically conductive type IV pili, or ‘nanowires’^48^ that facilitate DEET and allow long range charge transfer through an established biofilm of cells attached to a surface. Our work describes the role of surface area, conductivity, and catalytic effect in MFC anodes. Volumetric power density (*P*_V_) from MFCs using carbon foam anodes was doubled to 265 ± 12.1 mW m^–3^ by coating with a pristine graphene/PD (Gr–PD) based ink, and the enhanced surface from composite Gr–PD aerogel anodes increased *P*_V_ 13-fold to 3.51 ± 0.50 W m^–3^, closer to our benchmark provided by carbon fibre as an anode material (5.37 ± 1.16 W m^–3^). As a practical application, we show that a circuit of 10 single chamber MFC devices operated with Gr–PD aerogel anodes with a total volume of 1.32 cm^3^ generated 4.19 μW of power, sufficient to run a digital clock. In addition, we show that a Gr–PD ink coating is able to impart catalytic activity onto a standard marine grade stainless steel mesh (SS), showing feasibility as a cost-effective Pt-free air cathode. Finally, we demonstrate a fully pristine graphene-enabled MFC by integrating our pristine graphene-based anode aerogel and air cathode in a single chamber MFC, paving the way to a cost-effective, environmentally friendly energy source.

### Fabrication and characterisation of aerogel anodes

We prepared low density, highly porous aerogel anodes using a biocompatible and biodegradable non-conductive polymer, carboxymethylcellulose sodium salt (CMC) as a scaffold material.^49^ To establish the effect of conductivity and catalysis on MFC performance we created four aerogels by freeze drying (see Experimental for more details): a control CMC aerogel (A-CMC), a CMC–graphene aerogel (A-CMC–Gr), a CMC–PD aerogel (A-CMC–PD), and a CMC–Gr–PD aerogel (A-CMC–Gr–PD). For A-CMC, a CMC–water precursor solution was prepared, to which graphene flakes (Gr flakes, Cambridge Nanosystems, thickness ∼5 nm and lateral size ∼1 μm, ESI Fig. 1a†) were added to make A-CMC–Gr. PD (10% v/v) was added to make A-CMC–PD, and both Gr flakes and PD were added to make A-CMC–Gr–PD. The aerogels were then characterised by Raman spectroscopy, electrochemical impedance spectroscopy (EIS), mercury porosimetry and scanning electron microscopy (SEM).

**Fig. 1:**
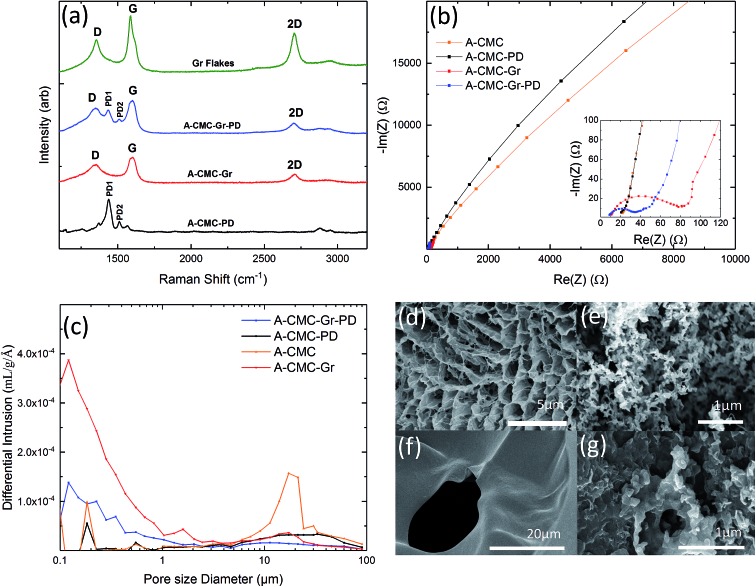
(a) Raman spectra of Gr flakes (green), A-CMC–PD aerogel (black), A-CMC–Gr aerogel (red), and A-CMC–Gr–PD aerogel (blue). (b) Nyquist curves of the electrochemical impedance spectra for each aerogel anode material. (c) Pore size distribution for A-CMC (orange), A-CMC–Gr (red), A-CMC–PD (black) and A-CMC–Gr–PD (blue). (d) SEM showing micrometre sized pores in the A-CMC, (e) A-CMC–Gr, (f) A-CMC–PD and (g) A-CMC–Gr–PD aerogels.

Fig. 1a plots the Raman spectrum of the Gr powder (green curve), A-CMC–Gr–PD (blue curve), A-CMC–Gr (red curve), and A-CMC–PD (black curve). The Raman spectrum, taken at 514 nm of the A-CMC–PD (black curve) aerogel exhibits several peaks which are typically assigned to PD's carbon stretching vibrations.^50^,^51^ The two more prominent peaks, found at ∼1435 cm^–1^ (PD1) and ∼1508 cm^–1^ (PD2), are assigned to the asymmetric *C*_α_ = *C*_β_ stretching and symmetric *C*_α_ = *C*_β_ (–O) stretching vibrations respectively.^50^,^51^ The red, blue and green curves present a G peak which corresponds to the E_2g_ phonon at the Brillouin zone centre in graphene, while the D peak (red, blue and green curves) is due to the breathing modes of carbon sp^2^ atoms and requires a defect for its activation.^52^–^54^ The 2D peak (red, blue and green curves) is the D peak overtone and is usually composed of a single Lorentzian in single layer graphene.^55^ A single Lorentzian fit of the 2D peak indicated that the graphene in our aerogels was comprised of electronically decoupled graphene layers. The analysis of the dispersion of the G peak (Disp(G)) (see Experimental) allows one to distinguish between in-plane defects and edge defects in graphene. The Disp(G) (0.07 ± 0.03 cm^–1^ nm^–1^) for each of the aerogels with graphene flakes (red, blue and green curves) indicates that the D peak originated from defects in the basal plane of the graphene in addition to defects along the flake edges.^52^,^56^ The PD1 and PD2 peaks were also found alongside the G and D peaks in the spectrum of the A-CMC–Gr–PD aerogel indicating the presence of both PD and graphene.

The Nyquist plots of the electrochemical impedance spectra (EIS) before the addition of *R. palustris* cells were used to determine the charge transfer resistance (*R*_ct_) of each aerogel. Nyquist plots are generated by plotting the imaginary impedance Im(*Z*) *versus* the real impedance Re(*Z*) for each aerogel, and show a semicircle at high frequencies, where the system is under kinetic control (*i.e.* when the electrochemical reaction is slow and local concentration gradients of electroactive species, such as ions and molecules, are negligible), followed by a straight line at low frequencies, where the system is under a diffusive controlled regime (*i.e.* when the electrochemical reaction is limited by the mass transport of the electroactive species that enter or leave the electrode surface).^40^ This can be described by an equivalent circuit model (ECM) (see ESI Fig. 2†). The series resistance (*R*_s_) combines the ionic resistance of the electrolyte, the intrinsic substrate resistance and the contact resistance, and it is defined as the value where the semicircle intercepts the real impedance (Re(*Z*)) axis.^57^ The high frequency semicircle can be described by the double layer capacitance (*C*_d_), and the charge transfer resistance (*R*_ct_). The diffusive regime is modelled by the Warburg resistance (*Z*_W_), which describes the frequency dependence of the ion transport to the electrode. In cases where the *R*_ct_ is sufficiently high and the diffusive regime is not reached, *Z*_W_ is set to zero^40^ (ESI Table 1†). Fig. 1b shows the Nyquist plots for each aerogel sample. In the case of A-CMC (orange curve) and A-CMC–PD (black curve) the *R*_ct_ is ∼101 kΩ and ∼176 kΩ respectively, with the single semicircle indicating that the electrochemical reaction is kinetically controlled, hindering the process of electron transfer and indicating a resistive behaviour of the materials.^36^ The lower *R*_ct_ for A-CMC–Gr (∼46.2 Ω) (red curve) and A-CMC–Gr–PD (∼21.0 Ω) (blue curve) suggests that the addition of Gr flakes is primarily responsible for decrease in *R*_ct_, while PD likely helps to bridge between the conductive graphene flakes, improving *R*_ct_ further. On the other hand, we noticed the absence of a diffusion element in the A-CMC–PD electrode, which indicates a slower ion transfer process. Therefore we can conclude that the addition of graphene flakes can improve electron transfer in the anode aerogels.

The pore size distribution of the aerogels was estimated by mercury porosimetry (see Experimental for more details). Fig. 1c shows the differential intrusion as a function of the pore size diameter for all the aerogels. In the case of A-CMC aerogel (orange line) we noticed a predominant peak in pore size at ∼17 μm, while the A-CMC–PD aerogel (red line) showed a broader pore size distribution between ∼10 to 90 μm. In the case of the A-CMC–Gr (red line) and A-CMC–Gr–PD (blue line) aerogels, the pore distribution shifted down to a 0.1–1 μm range, which might be attributed to graphene flakes blocking the pores which are >10 μm and thus creating smaller cavities throughout the aerogel which increases the resulting surface area. The corresponding calculated surface area (*S*_a_) was 3.9 m^2^ g^–1^ for the A-CMC–PD aerogel and 7.1 m^2^ g^–1^ for A-CMC, while *S*_a_ increased to 20.2 m^2^ g^–1^ and 8.2 m^2^ g^–1^ for A-CMC–Gr and A-CMC–Gr–PD respectively, with the addition of graphene flakes. We suspect that the surface areas are indeed much higher (*i.e.* 50–100 m^2^ g^–1^) than those calculated. However, a collapse of the aerogels due to increasingly high mercury pressure (∼400 psi) is known to affect soft foams analysed with mercury porosimetry^58^ by altering the statistics of the smallest pores (∼10 nm)^58^,^59^ due to their collapse. Thus their surface area contribution is masked. Scanning electron microscopy (SEM) of the aerogels was used to corroborate the results on the pore size distribution of the aerogels. Arrays of pores >1 μm in diameter were visible in the SEM images of the A-CMC (Fig. 1d), while in A-CMC–Gr (Fig. 1e) graphene flakes were entwined in fine porous structures with <1 μm in diameter. SEM images of A-CMC–PD (Fig. 1f) showed a smooth structure, with larger pores ∼20 μm in size comparable to the pore distribution (∼10 to 90 μm) determined by mercury porosimetry. Fig. 1g shows the graphene flakes blocking the majority of macropores (>1 μm in diameter) in A-CMC–Gr–PD, while pores <1 μm in diameter are still observable, thus confirming the role of the large flakes (∼1 μm) of graphene as a bridging material across the porous CMC scaffold.

### Fabrication and characterization of stainless steel cathodes and carbon foam anodes

In order to establish the effect of enhancing anode conductivity and surface area on MFC performance, we compared the aerogel anodes with a conductive graphene coated carbon foam (CF-Gr–PD) (see Experimental). This was prepared using a low surface area (∼3 m^2^ g^–1^) carbon foam (CF) coated with a graphene–PD ink (Gr–PD–IPA) formulated by liquid phase exfoliation (LPE) (see Experimental). Isopropyl alcohol (IPA) was used as the solvent for this ink as the low surface tension (∼29 mN m^–1^) helps to transport the graphene flakes by capillary action around the porous structure. Additionally, to investigate the catalytic activity of a graphene–PD coating as a Pt-free air cathode we coated a standard SS mesh with a graphene–PD ink (Gr–PD–W), by vacuum filtration (see Experimental), to make a graphene coated stainless steel (SS–Gr–PD) cathode. Water was chosen as the solvent for this ink as organic solvents such as IPA will dissolve the nitrocellulose membrane used in the vacuum filtration. The optical absorption spectra of the Gr–PD–W and Gr–PD–IPA inks (ESI Fig. 3†) were used to estimate the flake concentration^60^,^61^ *c*, obtaining *c*_Gr–PD–W_ ∼ 0.18 mg ml^–1^ and *c*_Gr–PD–IPA_ ∼ 0.08 mg ml^–1^. Atomic force microscopy (AFM) statistics showed a thickness of ∼6 nm (ESI Fig. 1a†) and lateral size ∼135 nm (ESI Fig. 1b†) for the Gr–PD flakes. Rheological measurements determining viscosity (*η*), surface tension (*γ*), and density (*ρ*) for the two inks showed that *η*_Gr–PD–W_ ∼ 0.89 mPa s, *γ*_Gr–PD–W_ ∼ 70 mN m^–1^, *ρ*_Gr–PD–W_ ∼ 1.02 g cm^–3^; *η*_Gr–PD–IPA_ ∼ 2.5 mPa s, *γ*_Gr–PD–IPA_ ∼ 27 mN m^–1^, *ρ*_Gr–PD–IPA_ ∼ 0.785 g cm^–3^, consistent with previous reports.^62^–^64^

**Fig. 2:**
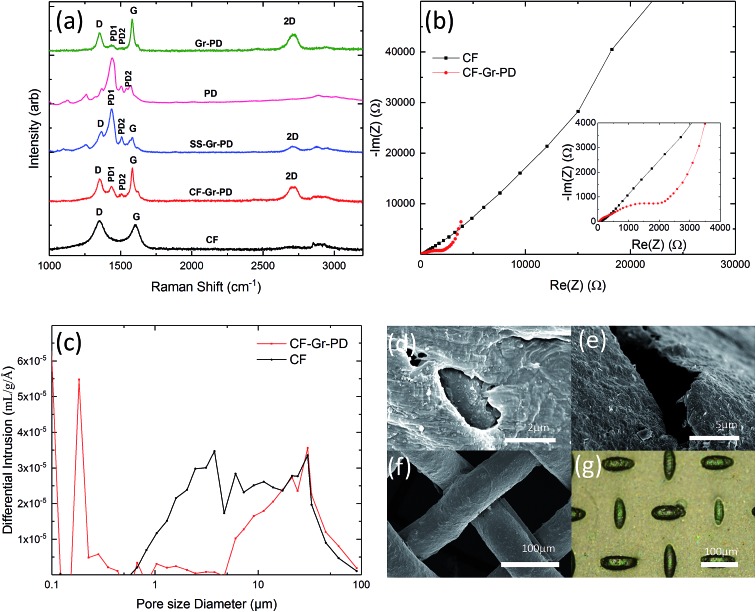
(a) Raman spectra of Gr–PD–IPA flakes (green), PD (pink), CF (black), CF-Gr–PD (red), and SS–Gr–PD (blue). (b) Nyquist curves of the electrochemical impedance spectra for the CF and CF-Gr–PD anodes. (c) Pore size distribution for the CF (0.0140 g) (black) and CF-Gr–PD (0.0120 g) (red) anodes. SEM images of the (d) CF, (e) CF-Gr–PD, and (f) SS anode, (g) bright field optical microscopy image of the SS–Gr–PD.

Raman spectroscopy was also used to characterize the quality of the cathode and anodes. Fig. 2a shows the Raman spectra (acquired at 514 nm) of the Gr–PD flakes (green curve) (which show the Raman fingerprint of the Gr–PD–W flakes as discussed in ESI Fig. 4†), the PD (pink curve), the SS–Gr–PD (blue curve), the CF-Gr–PD anode (red curve) and the CF anode (black curve). Besides the PD1 and PD2 peaks at 1435 cm^–1^ and 1508 cm^–1^, the SS–Gr–PD cathode (blue curve) and CF-Gr–PD anode (red curve) have the typical D, G and 2D peaks of graphene as described in the previous section which are in line with the spectra of Gr–PD–IPA flakes (green curve). The blue curve showed a combination of both Gr–PD and PD spectra, while the red curve brings additional features to the CF anode (black curve) where the absence of a distinct 2D peak and the G peak position Pos(G) ∼ 1600 cm^–1^ indicated the more defective nature of the CF.^52^,^65^,^66^ For disordered carbons Pos(G) increases linearly as the excitation wavelength decreases from infrared to ultraviolet, therefore Disp(G) increases with disorder.^45^ For carbon systems which have a large number of structural defects Disp(G) > 0.1 cm^–1^ nm^–1^.^55^ We attribute the D peak intensity predominantly to the edges of our submicrometer flakes, rather than to structural defects within the flake, given a Disp(G) (0.011 ± 0.003 cm^–1^ nm^–1^) lower than that expected for disordered carbon.^52^,^56^ Therefore, there was a lack of large structural disorder within our flakes and scattering only occurred at the edges of the flakes in an otherwise defect-free sample.^49^

Nyquist plots of EIS (Fig. 2b) showed that the *R*_ct_ of CF-Gr–PD anode decreased with respect to that of the CF anode from 41.4 kΩ to 0.930 kΩ. (ECM, ESI Fig. 2†), indicating that addition of the Gr–PD–IPA flakes results in a decrease in *R*_ct_. The pore size distribution and specific surface area of the CF-Gr–PD and CF anodes were determined using mercury porosimetry. Fig. 2c shows a broad pore size distribution for the CF and CF-Gr–PD anodes between ∼1 and 100 μm. The specific surface area was calculated (see Experimental) and was similar for both CF (3.7 m^2^ g^–1^) and CF-Gr–PD (2 m^2^ g^–1^) anodes. SEM of the CF and CF-Gr–PD anodes showed average pore sizes of ∼2 μm (Fig. 2d) and ∼5 μm (Fig. 2e) in diameter respectively, matching with the porosimetry results. Fig. 2f, acquired by SEM, shows the microstructure of the SS mesh, and Fig. 2g shows the SS–Gr–PD cathode, by optical microscopy, confirms the presence of a Gr–PD continuous film in between the SS mesh wires.

### Bioelectrochemical characterization of aerogel anodes

**Fig. 3:**
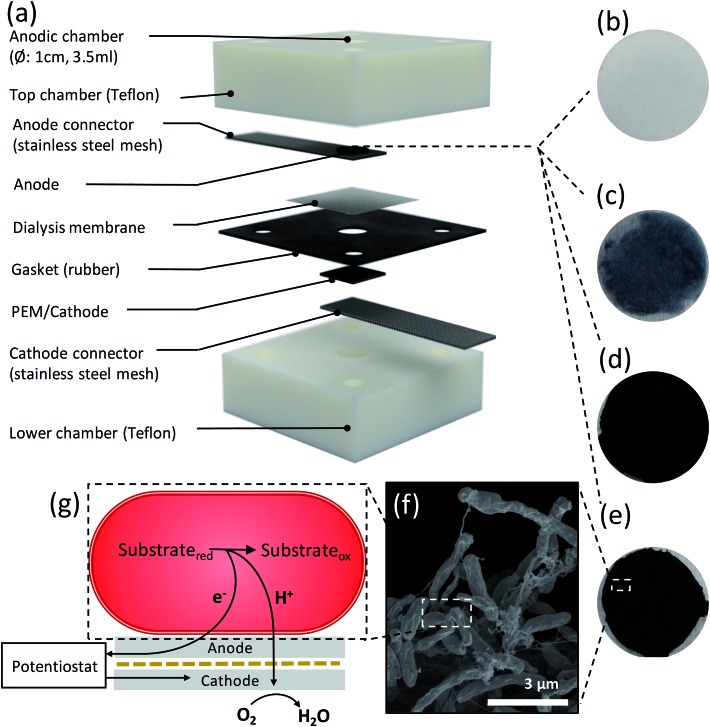
(a) Diagram of the MFC chamber components. Photographs of the aerogel disks: (b) a A-CMC, (c) PD A-CMC–PD, (d) A-CMC–Gr and (e) A-CMC–Gr–PD. (f) SEM micrograph of *R. palustris* biofilm on the anode. (g) A simplified diagram illustrating the oxidation of substrate metabolites to provide reducing power that is transferred to the anode through a circuit to the cathode *via* a potentiostat. To balance charges, mass transport of protons also generated from metabolism occurs through a proton exchange membrane to the air exposed catalytic side of the cathode membrane, where they combine with electrons and atmospheric oxygen to form water.

To compare the bioelectrochemical performance of the aerogel and CF based anodes we designed a single chamber MFC (Fig. 3a) for repeated and reliable experimental use. The MFC electrode components were assembled in a stack made in descending order of the anodic aerogel (Fig. 3b–e), an SS anode connector, a dielectric dialysis membrane layer with pore size sufficient to block bacterial cells, a Nafion® proton exchange membrane (PEM), with the lower side coated with a conductive and catalytic carbon–Pt surface, and an SS cathode connector. The electrode materials stack was clamped between two Teflon® blocks each with 4 ml cylindrical chambers drilled through them (see Experimental for dimensions), and a rubber gasket seal. The upper chamber was inoculated with *R. palustris* at an optical density (measured at 600 nm) OD_660_ = 3.0 without stirring, to encourage cells to form an electroactive biofilm on the anode surface (as shown by the SEM image in Fig. 3f). Fig. 3g shows a basic schematic of MFC function.

**Fig. 4:**
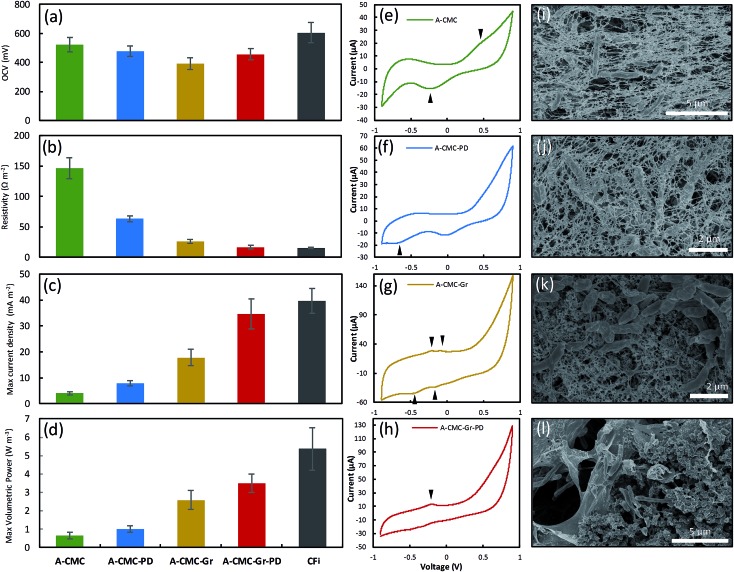
(a) Comparison of open circuit potentials, (b) surface resistances *R*_sur_, (c) maximum current densities, and (d) maximum volumetric power outputs from stainless steel mesh anodes mounting A-CMC, A-CMC–PD, A-CMC–Gr, A-CMC–Gr–PD aerogels, and carbon fibre (CFi), respectively. Error bars show the standard error, for the aerogels of *n* = 10, and for CFi *n* = 3. Cyclic voltammograms of the stainless steel anode with (e) A-CMC, (f) A-CMC–PD, (g) A-CMC–Gr, and (h) A-CMC–Gr–PD aerogels on top of stainless steel mesh. Scan speed was 1 mV s^–1^ between –0.9 and +0.9 V. Each CV trace is a single representative sample, arrows show discernible oxidation and reduction peaks. Sem images of the aerogel anodes show *R. palustris* cells embedded in the (i) A-CMC, (j) A-CMC–PD, (k) A-CMC–Gr, and (l) A-CMC–Gr–PD aerogels.

We used linear sweep voltammetry (LSV) to calculate polarization and power curves of the aerogel anodes by applying a linear sweep potential from the open circuit voltage (OCV) to 0 V. The OCV (Fig. 4a), surface resistance (calculated by the gradient of the *I*–*V* polarization curve) *R*_sur_ (Fig. 4b), maximum current density, *I*_D_ (Fig. 4c) (normalized to projected cathode surface area^67^), and maximum volumetric power output, *P*_V_ (Fig. 4d) (normalised to anode volume^68^ and calculated *via* Ohm's law^69^) for the A-CMC, A-CMC–PD, A-CMC–Gr, and A-CMC–Gr–PD aerogels were determined and compared with the benchmark anode material, carbon fibre (CFi). After inoculation for 12 hours with *R. palustris*, the stable OCV of A-CMC, A-CMC–PD, A-CMC–Gr and A-CMC–Gr–PD reached 522 ± 47.7 mV, 476 ± 35.0 mV, 391 ± 38.9 mV, and 456 ± 38.0 mV respectively. The MFC using A-CMC aerogel gave *I*_D_ and *P*_V_ of 4.04 ± 0.618 A m^–2^ and 0.648 ± 0.178 W m^–3^ respectively (Fig. 4c and d). Considering that CMC is an electrical insulating polymer which gives A-CMC a *R*_sur_ of 146 ± 17.3 Ω m^–2^ (the highest of the aerogel anodes, Fig. 4b), A-CMC may favour the formation of a conductive biofilm in contact with the SS anode connector which could result in giving the A-CMC some conductive properties. Using A-CMC–PD as an anode reduced *R*_sur_ to 63.5 ± 5.09 Ω m^–2^, while *I*_D_ and *P*_V_ were marginally increased over A-CMC to 7.91 ± 0.923 A m^–2^ and 1.01 ± 0.178 W m^–3^ respectively (Fig. 4c and d). Using A-CMC–Gr further reduced *R*_sur_ to 26.3 ± 3.24 Ω m^–2^ and increased *I*_D_ and *P*_V_ to 17.9 ± 3.09 A m^–2^ and 2.59 ± 0.514 W m^–3^, which is a 4-fold increase over the A-CMC and A-CMC–PD aerogels. We noticed that *R*_sur_ was lowest in A-CMC–Gr–PD at 16.7 ± 2.86 Ω m^–2^, and *I*_D_ and *P*_V_ increased to 34.61 ± 5.84 A m^–2^ and 3.51 ± 0.504 W m^–3^. These results are consistent with the trend of *R*_ct_ shown previously by EIS indicating once more that the addition of graphene flakes helps to improve electron transfer between the anode and biofilm, resulting in improved *P*_V_ and *I*_D_ in our MFC. Notably, whilst *P*_V_ increased by 36% using the A-CMC–Gr–PD over the A-CMC–Gr aerogel, inclusion of PD doubled *I*_D_; this is likely to be due to the highly conductive nature of the PD which allows current to flow easily though the aerogel matrix to the SS anode connector. This may provide conductive bridges between the graphene flakes,^70^ thereby reducing *R*_sur_ and improving charge transfer. Moreover, while it was observed that the fragile A-CMC–Gr aerogels partially disintegrated in the aqueous cell culture medium, the presence of PD in A-CMC–Gr–PD improved the structural robustness of the aerogels. A-CMC–Gr–PD comes closer to the CFi, in terms of power output (5.37 ± 1.16 W m^–3^), and has similar surface resistance (15.3 ± 1.21 Ω m^–2^), however the higher OCV of CFi (605 ± 70.3 mV) may have facilitated higher current (39.7 ± 4.86 A m^–2^) due to the unbroken conductive CFi connection, as opposed to discontinuous pristine graphene flakes.

Cyclic voltammetry (CV) can be used to gain a qualitative insight into the redox mechanisms used to transfer electrons between the cell and the anode and thus give information on pristine graphene's catalytic properties (*i.e.* efficiency of EET or charge transfer).^71^Fig. 4e–h shows representative cyclic voltammograms of MFC devices operated with each aerogel colonized by *R. palustris* taken at 1 mV s^–1^ between –900 mV and 900 mV after 72 hours in the devices. A broad oxidation–reduction peak pair at ∼360 mV and ∼–200 mV from A-CMC are most likely a result of the interaction between cells and the stainless steel anode connector,^72^ and confirm the establishment of an electroactive biofilm on the anode.^73^ The addition of PD to CMC (A-CMC–PD) did not change the CV profile significantly (Fig. 4f), other than the emergence of a small reduction peak at –640 mV. The addition of Gr flakes to A-CMC–Gr (Fig. 4g) increased the current range in response to the voltage scan, and revealed two pairs of oxidation–reduction peaks at –230 mV and –420 mV, and –100 mV and –160 mV. These peaks are similar to those reported for *R. palustris* on carbon paper,^46^ and are in accordance with previous studies that suggest graphene has a more significant effect on EET to enhance MFC current generation rather than *via* interaction with excreted mediators.^33^,^74^ The emergence of these peaks indicates a favourable interaction between at least two extracellular redox mechanisms with graphene, with a low degree of separation between oxidation and reduction peaks being characteristic of an easily reversible reaction with enhanced charge transfer.^75^ When both graphene and PD were incorporated into the A-CMC–PD–Gr aerogel anode (Fig. 4h), the oxidative peak at –230 mV was more prominent, and the neighbouring oxidation peak at –100 mV was no longer visible, suggesting that one redox mechanism with a lower activation energy for charge transfer is being favoured. Our results show similar profiles to other organisms with more extensively characterized metal reducing outer membrane cytochromes (Omc) such as OmcA and the Mtr pathway from *Shewanella oneidensis*.^75^ However *R. palustris* homologs to OmcA have low genetic and structural similarity^48^ and *R. palustris* is known to have other important mechanisms of both oxidising and reducing its surroundings such as the phototrophic iron oxidation (Pio) pathway.^76^ CV data show that graphene enhances DEET from *R. palustris* in a MFC. However, further work is required to elucidate the molecular basis of the precise redox active mechanisms acting in synergy with graphene.

### SEM of colonized aerogel anodes

SEM imaging of the aerogel anodes after their use in the MFC (Fig. 4i–l) showed cells embedded in the aerogel material with an extremely high level of cell to anode contact. SEM of the A-CMC and A-CMC–PD showed networks of anode aerogel material surrounding and in contact with cells (Fig. 4i and j). SEM of the A-CMC–Gr and A-CMC–Gr–PD aerogels (Fig. 4k and l) also showed an interconnected structure of graphene and polymer matrix. We noticed that unlike some silica based aerogels that maintain their structure after re-hydration,^77^ our CMC based aerogels visibly contract upon contact with cell media. Capillary action and contraction of the super-dehydrated material may help incorporate and immobilize cells within the microstructure of the aerogel and maximize anode to cell contact, which could potentially improve EET.

### Bioelectrochemical characterization of graphene coated carbon foam anodes

**Fig. 5:**
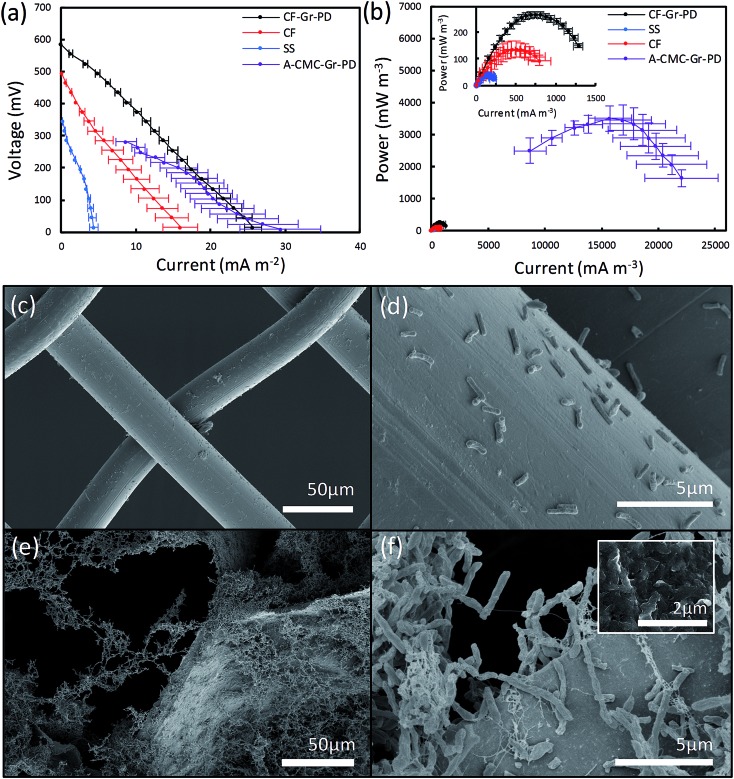
(a) Polarization curves and (b) power curves for MFC devices operated with SS (blue), CF (red), CF-Gr–PD (black), and A-CMC–Gr–PD (purple) anodes respectively. Inset to (b) are the power curves of SS, CF, and CF-Gr–PD for clarity. Platinum coated carbon paper (CP-Pt) was used as open air cathode. The volumetric current and power output is expressed based on the geometrical size of the anode chamber. For SS *n* = 6, for CF and CF-Gr–PD *n* = 9, and for A-CMC–Gr–PD *n* = 10. SEM images of (c) SS with *R. palustris* cells and (d) SS with a graphene coating and cells. SEM images of (e) CF with *R. palustris* cells and (f) CF with *R. palustris* cells with graphene, inset: further magnification showing graphene flakes.

We assessed the influence of surface area on the MFC anodes by acquiring the polarisation and power curves from linear sweep voltammetry from MFC devices equipped with CF anodes and the SS anode connector alone (used as control), as done for the aerogels. The *I*_D_ and *P*_V_ can be identified from the polarization curves (Fig. 5a) and power curves (Fig. 5b) of the CF (inset red), CF-Gr–PD anodes (inset black) and SS (inset blue), with the A-CMC–Gr–PD curves shown to illustrate the order of magnitude difference in power output most likely due to surface area. OCV for the CF-Gr–PD anode was measured as 669 ± 6.92 mV, compared to 480 ± 30.9 mV with CF and 392 ± 21.4 mV with just SS at the anode. We also obtained similar values of *R*_sur_ for CF and CF-Gr–PD at 30.8 ± 5.30 Ω m^–2^ and 23.7 ± 3.48 Ω m^–2^ respectively, and lower than SS at 86.4 ± 25.5 Ω m^–2^. The SS alone at the anode yielded *I*_D_ and *P*_V_ of 4.47 ± 1.06 mA m^–2^ and 43.6 ± 10.1 mW m^–3^ respectively. *I*_D_ and *P*_V_ of CF-Gr–PD were 25.7 ± 1.113 mA m^–2^ and 265 ± 12.1 mW m^–3^, both nearly 2-fold higher than *I*_D_ and *P*_V_ of CF at 15.9 ± 2.80 mA m^–2^ (*p* = 0.020) and 138 ± 28.2 mW m^–3^ (*p* = 0.007) respectively (ESI Table 2†). Since the CF and CF-Gr–PD have similar surface area, these results indicate that the conductive graphene coating improves the MFC performance resulting in higher *P*_V_, *I*_D_, and lower *R*_sur_. Furthermore the positive effect of the anode surface area on MFC performance can be inferred as *P*_V_ increased 13-fold from 0.265 ± 0.0121 W m^–3^ from the low surface area (∼3 m^2^ g^–1^) CF-Gr–PD anodes to 3.51 ± 0.504 W m^–3^ from the A-CMC–Gr–PD aerogel anodes with a surface area of ∼10 to 20 m^2^ g^–1^. This clearly indicates that increasing anodic surface area is a key factor to improve MFC performances.

### SEM of biofilms on carbon foam and steel anodes after use in MFC

In order to visualize the distribution of the *R. palustris* biofilms on the SS, CF, and CF-Gr–PD anodes after operations, the anodes were removed from the MFC and prepared for SEM imaging after performing the bioelectrochemical measurements. The SS anode connector (Fig. 5c and d) showed a very sparse presence of microbial cells, suggesting that these were more weakly attached to the smooth, convex surface than to the rougher surface of CF (Fig. 5e) and CF-Gr–PD anodes (Fig. 5f). Graphene flakes were visible on the surface of CF-Gr–PD (Fig. 5f inset). Whereas mainly single cells were visible attached to the smooth surface the SS wires, more extensive networks of cells with many cell to cell interactions were visible in the CF biofilm (Fig. 5f). Conductive type IV pili are thought to be an essential element of electrogenic and conductive bacterial biofilms,^10^,^12^ and *R. palustris* has previously been shown to produce conductive filamentous structures such as pili or ‘nanowires’.^48^ Here at least some of the filamentous structures visibly connecting cells to each other and to the surface of the anode were likely to be conductive pili that may play an important role in DEET.

### Graphene ink modified stainless steel cathode

**Fig. 6:**
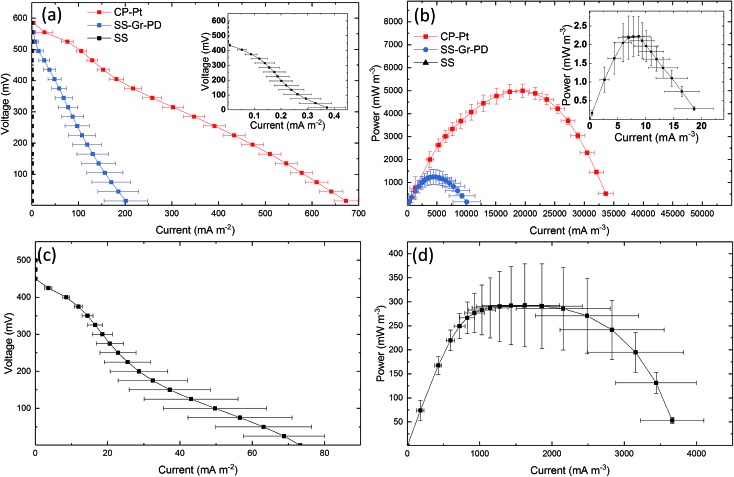
(a) Polarization and (b) power curves using SS (black) (inset), SS–Gr–PD (blue), and CP-Pt (red) air cathodes. Carbon fibre (2 g) was used in the anodic chamber, *n* = 6 for each sample, and error bars show the standard error. (c) Polarization curve and (d) power curve for MFCs using graphene at both electrodes, with A-CMC–Gr–PD in the anodic chamber and SS–Gr–PD as an air cathode, *n* = 3 and error bars show the standard error.

To establish the effect of PD and graphene flakes as a cathode we compared the MFC performance of devices configured with plain SS, SS–Gr–PD, and an industry standard platinum coated carbon paper (CP-Pt) at the cathode.^11^ A carbon fibre anode was used to verify the catalytic function (*i.e.* in this case to facilitate ORR) of pristine graphene with the SS–Gr–PD electrode. Fig. 6a and b show the polarization and power curves for each of the cathodes (see Experimental). SS–Gr–PD (blue curve) as a cathode yielded *I*_D_ and *P*_V_ of 0.172 ± 0.0378 A m^–2^ and 1.04 ± 0.252 W m^–3^ respectively, which are 500-fold greater than those achieved with SS (black curve) at the cathode (0.000373 ± 0.000103 A m^–2^ and 0.00228 ± 0.000552 W m^–3^) (Fig. 6a and b, insert, *p* = 0.011) and is only slightly lower than what was achieved when using a CP-Pt cathode (red curve) (*I*_D_ ∼ 0.672 ± 0.152 A m^–2^ and *P*_V_ ∼ 5.01 ± 0.302 W m^–3^). We attribute the improved performance of the SS–Gr–PD to the catalytic properties of pristine graphene flakes on the SS mesh facilitating the ORR.^7^,^8^ Finally, the combination of a A-CMC–Gr–PD aerogel anode with a SS–Gr–PD cathode yielded an *I*_D_ of 0.0753 ± 0.739 A m^–2^ (Fig. 6c) and *P*_V_ of 0.390 W m^–3^ (Fig. 6d), demonstrating the feasibility of Pt-free all-graphene catalysed MFCs.

### A digital clock powered by graphene based MFCs

**Fig. 7:**
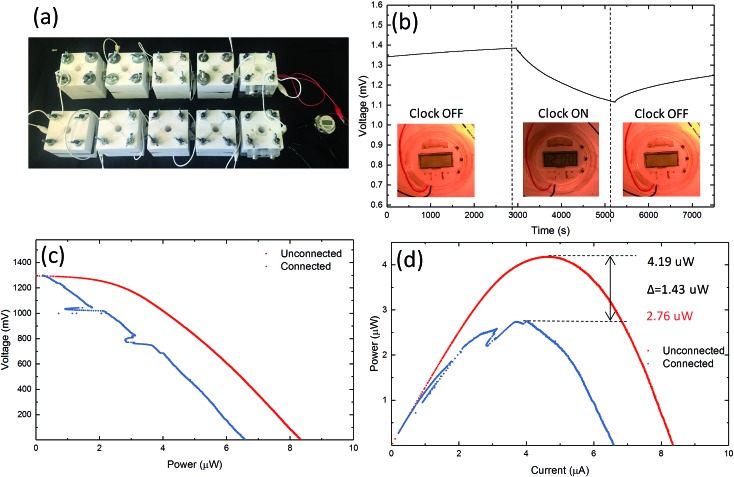
(a) Photograph 10 MFCs configuration powering clock, (b) chronopotentiometry showing effect of clock being connected. (c) Polarization and (d) power curves of MFCs with the clock absent (red) and connected (blue).

We tested the feasibility of graphene based MFCs to power small electrical devices, such as a digital clock. A circuit of 10 MFC chambers using A-CMC–Gr–PD anodes and Nafion® carbon–Pt cathodes was connected, with two series of five MFCs in parallel (Fig. 7a). This configuration was chosen to reach sufficient voltage for the digital clock to operate correctly. The MFCs produced 4.19 μW at 1.29 V and were able to power a clock successfully. Chronovoltammetry (Fig. 7b) showed a steady potential drop from 1.38 to 1.10 V in the circuit when the clock was connected. This was potentially due to an effective internal anode–cathode short circuit caused by faster oxidation–reduction kinetics at the anode than the cathode.^78^,^79^ Polarization (Fig. 7c) and power curves (Fig. 7d) from data before (red) and after (blue) the clock was connected indicate *I*_D_ and *P*_V_ decreasing from 8.3 to 6.5 μA and from 4.19 to 2.76 μW, respectively. This application demonstrates how graphene based MFCs are able to produce sufficient power to run commercial electronic devices such as those that may be found in wearable technology or low powered sensors.^28^ Our results demonstrate the viability of graphene based electrodes as efficient, cost-effective, biocompatible, environmentally-friendly and platinum-free anodes and cathodes for MFCs. This represents a disruptive step change in the manufacturing, cost, accessibility and sustainability of MFCs, paving the way, for example, to more accessible energy sources enabling democratisation of energy supply with important impacts in many aspect of our society from medicine to energy and consumer electronics.

## Conclusions

In this study we demonstrated the three-fold advantage of graphene based aerogels towards enhancing the efficiency of MFC electrodes. First, we demonstrated the effectiveness of unmodified, pristine graphene composite aerogels. Second, we showed that a pristine graphene coating can enhance EET compared to standard carbon anodes. Third, we reveal that pristine graphene can catalyse the cathodic ORR as well as anodic EET. We provide a direct demonstration of graphene enabled MFCs powering a commercial digital clock, illustrating the possibility of MFC in power demanding applications such as micro-electronics, beyond the already existing application ranges of sustainable electricity production, treatment of municipal waste water streams and biosensors.^26^,^80^ Finally, we demonstrate for the first time an entirely graphene catalysed MFC.

## Experimental

### Ink production

An isopropanol (IPA) based ink (Gr–PD–IPA) was prepared by ultrasonication (Fisherbrand FB15069, Max power 800 W) of graphite flakes (Sigma Aldrich) for 9 hours in IPA with PD (1% v/v). Additionally, a water based graphene ink (Gr–PD–W) was prepared by ultrasonication of graphite flakes for 9 hours in deionized water with PD (1% v/v) (Sigma 739316) to produce graphene flakes. In both ink preparations, the small amount of PD acts as a stabilisation agent and conductivity enhancer. The stabilization mechanism is due firstly to the π–π interaction between the graphene sheets and the backbone of the PEDOT and secondly the electrostatic repulsion between the negatively charged PSS.^81^ Both dispersions were then centrifuged (Beckman Coulter Proteomelab XL-A, with a SW 32 Ti swinging bucket rotor) at 5 k rpm for 1 hour to remove thick (>10 nm) flakes. After centrifugation, the top 70% of each dispersion was collected.

### Optical absorption spectroscopy

The graphene flakes concentration was estimated using optical absorption spectroscopy^61^,^62^,^82^ *via* the Beer–Lambert law expressed in the formula *A* = *αcl*, where *α* [L g^–1^ m^–1^] is the absorption coefficient, *c* [g L^–1^] is the concentration and *l* [m] is with the beam path length. The Gr–PD–W and Gr–PD–IPA inks were diluted 1 : 10 with water–PD and IPA–PD respectively. Assuming *α* ∼ 1390 L g^–1^ m^–1^ (ref. 61) and *α* ∼ 2460 L g^–1^ m^–1^ (ref. 82) at 660 nm for Gr–PD–W and Gr–PD–IPA, respectively.

### Ink characterisation

The surface tension of the inks was measured using the pendent drop method (FTA1000B). The shape of the drop results from the relationship between the surface tension and gravity. The surface tension is then calculated from the shadow image of a pendent drop using drop shape analysis. A parallel plate rotational rheometer (DHR rheometer TA instruments) was used to evaluate the viscosity as a function of shear rate and the infinite-rate viscosities were determined for the Gr–PD–W and Gr–PD–IPA inks. Ink density was evaluated from a (Sartorius ME5) microbalance where the density is the mass per unit volume (*ρ* = *m*/*V*).

### Graphene anodes fabrication

A graphene coated carbon foam CF-Gr–PD anode was created by drop casting 3 ml of Gr–PD–IPA ink onto a conductive carbon foam anode (Conductive Foam Pad 40 × 40 mm, Maplin, UK). The low surface tension (29 mN m^–1^) of the Gr–PD–IPA ink transported the graphene flakes by capillary action around the porous structure. For the aerogel anode fabrication a CMC/water precursor for each aerogel was created by adding 5 mg ml^–1^ CMC (weight average molecular weight, MW ∼ 700.000, Sigma Aldrich 419338) to deionised water. Graphene powder (10 g L^–1^) (Cambridge Nanosystems) produced by cracking methane and carbon dioxide in a plasma torch was then added to the CMC/water solution for the A-CMC–Gr–PD and A-CMC–Gr aerogels while PD (10% v/v) (Sigma 739316) was added to the CMC/water solution for the A-CMC–Gr–PD and A-CMC–PD aerogels. Each sample solution (1 ml) was pipetted into an aluminium container and frozen at –10 °C allowing the growth of ice crystals to shapes the pore geometry of each structure. The samples were then placed into a freeze dryer (Telstar LyoQuest) to remove the ice crystals by sublimation under vacuum.

### Cell culture

Wild type *R. palustris* CGA009 was grown in minimal medium^83^ with 40 mM glycerol as the carbon source and 5 mM urea as nitrogen source in 0.5 litre sealed Schott bottles in a shaker incubator at 120 rpm and 30 °C under fluorescent lights. Cells in 50 ml of culture were collected by centrifugation at 4000 rpm and adjusted to OD_660_ = 3.0 for inoculation into the MFC devices, 4 ml per device.

### Graphene cathode construction

The cathodes were prepared by vacuum filtration transfer. First (∼4 ml) of the Gr–PD–W ink was diluted with deionized water at a ratio of 1 : 9 respectively and passed through a nitrocellulose membrane (100 nm pore size), hastened with the use of a Büchner flask attached to a vacuum pump. The graphene/PD film on the membrane was then transferred onto a stainless steel mesh with 0.2 mm spacing. After oven annealing (∼80 °C) the sample was placed in an acetone bath overnight in order to dissolve the nitrocellulose membrane and leave behind a graphene/PD film of thickness 635 nm (Bruker Dektak Stylus Profilometer) on the metal mesh (cathode SS–Gr–PD). SS–Gr–PD was then cleaned in acetone and isopropanol baths sequentially and then died in a nitrogen flux. The sheet resistance (*R*_s_) of each cathode was determined with a Jandel probe head in a 4-point probe configuration. The *R*_s_ of a Gr–PD film transferred onto glass is 3.7 kΩ □^–1^, significantly higher than the *R*_s_ of the SS cathode (<10 Ω □^–1^).

### Pore size distribution and surface area

The pore size distribution and specific surface area of the anodes were determined using a mercury intrusion technique (Micromeritics AutoPore IV 9500). Mercury was pushed into the sample from 6.9 kPa (1 psia) to a maximum pressure of 206 843 kPa (30 000 psia). The relationship between this pressure (*P*) and the pore diameter (*D*) can be found through the Washburn equation assuming the pores are cylindrical,^58^,^59^ *D* = –4*γ* cos(*θ*)/*P* where *γ* is the surface tension of mercury (485 mN m^–1^), *θ* is the contact angle (130°) acting along the parameter of the pore. The pore size diameter is plotted as a function of the differential intrusion (ml g^–1^ Å^–1^) which is found by dividing the incremental intrusion (ml g^–1^) by the difference in pore diameter (Å). The specific pore area (*A*) is then calculated (*A* = 4*V*/*D*) assuming a cylinder pore volume (*V* = π*D*2*h*/4) and open cylinder pore area (*A* = π*Dh*). For each specific surface area measurement the contribution from the macro (∼75 μm) and meso (∼1 μm) pores while the contribution from the micropores (∼10 nm) could not be determined as the foams collapsed at the higher pressures (∼400 psia), a known problem when examining soft foams with mercury porosimetry.^58^,^59^

### Electrochemical impedance spectroscopy (EIS)

EIS was conducted in a two-electrode setup. Each working electrode was an aerogel or foam attached directly to a stainless-steel mesh. The electrodes were separated by a filter paper (Millipore JVWP, 0.1 μm pore size) and pressed together between PTFE blocks. The cell was immersed in 1.0 M tetraethyl ammonium tetrafluoroborate (TEABF)/propylene carbonate (Sigma-Aldrich) non-aqueous electrolyte. EIS experiments were conducted using a BioLogic VSP-300 potentiostat, using an AC voltage of 0.2 V with 5 mV amplitude over a frequency range of 10 mHz to 10 kHz. All experiments were performed at room temperature.^40^

### Electrochemical measurements

The MFCs used in this study were based around the design shown in Fig. 1. The MFC system consisted of two blocks of Teflon® (60 × 60 × 30 mm) with drilled cylindrical channels (*Ø*: 13 mm, height 30 mm), between which were clamped in descending order: a marine grade stainless steel (Mesh Company Ltd, UK) anodic electrode connector, a dialysis membrane of 10 kDa pore size (Thermo), a Nafion® proton exchange membrane (Dupont) with a conductive and catalytic carbon–Pt coating facing into the air exposed cathodic chamber, and finally a second SS mesh. The top and bottom SS mesh layers served as contacts for the anode and cathode respectively. Carbon fibre and aerogel electrodes were placed over the anodic SS mesh with wet thickness of 4 mm and 1 mm respectively, and total geometrical area of 1.33 cm^2^. No electron shuttle mediator was used. 4 ml of adjusted cell culture was injected into the top anodic chamber and cells were allowed to settle and form a biofilm on the anode. To characterize the SS–Gr–PD cathodes, the MFC device was modified with a secondary channel (*Ø*: 6 mm) drilled perpendicular and intersecting midway to the anodic channel. The SS–Gr–PD cathode was placed at the end of this channel, with a total geometrical area of 0.283 cm^2^, and 2 g of carbon fibre (Carbonmods Ltd, UK) was used in the anodic chamber. Platinum coated carbon paper (CP-Pt) was used as a benchmark (hydrogen electrode/reformate cathode Alfa Aesar 45452). Bioelectrochemical characterisation was carried out with a PALM-SENS MultiEmstat 8-channel potentiostat. Following stabilisation at the open circuit potential, polarisation and power curves were calculated by Ohm's law *via* linear voltage sweeps scanning the applied voltage from its open circuit potential to 0 volts with a slow scan rate of 1 mV s^–1^ to avoid overpotential effects.^84^ Cyclic voltammetry measurements were also carried out at 1 mV s^–1^ between –900 mV and 900 mV after incubation for 3 days in the MFC.

### Raman spectroscopy

Raman spectra for the cathode and anodes were acquired with a Renishaw 1000 InVia micro-Raman spectrometer at 457, 514.5, and 633 nm and a ×20 objective, with an incident power of below ∼1 mW.^64^ The G peak dispersion is defined as Disp(G) = ΔPos(G)/Δ*λL*, where *λL* is the laser excitation wavelength. Raman spectra for the Gr–PD–IPA and Gr flakes were acquired on Si/SiO_2_ substrate while all other spectra were acquired on the anodes and cathodes directly.

### Scanning electron microscopy

Scanning electron microscopy images were taken with a high resolution Magellan 400L scanning electron microscope (SEM). The field emission gun was operated at an accelerating voltage of 5 keV and gun current of 6.3 pA. Images were obtained in secondary electron detection mode using an immersion lens and TLD detector. The biological samples were taken on a FEI Verios 460 scanning electron microscope. Samples were washed in distilled water, fixed in liquid ethane, and mounted on a liquid nitrogen cooled plate before freeze drying overnight and coating in 14 nm of iridium.

## Author contributions

T. C. and T. P. C. conceived and designed the experiments. T. C., T. P. C., P. B., F. T. and C. J. H. analysed the experimental data and interpreted the results. T. C. developed the inks and composites and performed UV-vis, rheometry, AFM, Raman and pendant drop analysis on the inks and conducted mercury porosimetry, SEM, optical microscopy on the carbon foam and aerogel composites. T. P. C. and D. L. S. curated *R. palustris* and prepared cell cultures, T. P. C. and P. B. designed and constructed the MFCs and performed electrochemical measurements. T. P. C. undertook SEM on biological samples while P. H. preformed EIS on the aerogel and carbon foam samples with the assistance of T. C. The manuscript was written by T. C., T. P. C., F. T. and C. J. H., in close consultation with other authors.

## Conflicts of interest

The authors have no conflict of interest.

## Supplementary Material

Supplementary information
