# Supplementary material for: Platinum-free, graphene based anodes and air cathodes for single chamber microbial fuel cells
Source: J Mater Chem A Mater. 2017 Nov 2;5(45):23872–86. doi: 10.1039/c7ta06895f (PMC5795293; doi:10.1039/c7ta06895f)
Supplement: Supplementary file 1 [file TA-005-C7TA06895F-s001.pdf]

Electronic Supplementary Material (ESI) for Journal of Materials Chemistry A.
This journal is © The Royal Society of Chemistry 2017

Platinum-free, graphene based anodes and cathodes for single chamber microbial fuel cells with *Rhodopseudomonas palustris*

Supplementary Information:

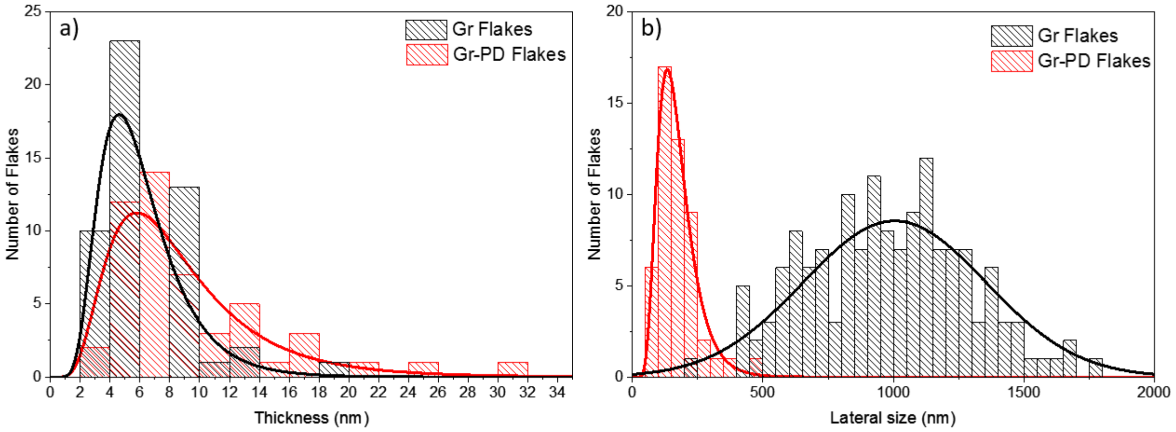

Sup. Fig 1: Flake thickness and lateral size distribution (a), for Gr flakes and (b) Gr-PD flakes. The average lateral size and thickness of the Gr-PD flakes prepared for the ink coatings and the CNS flakes used in the aerogels were estimated by atomic force microscopy (AFM). A Bruker Dimension Icon working in peakforce mode was used. The AFM statistics on the flake thickness (Supp. Fig. 2a) shows a log-normal distribution[1] peaking at 5 nm and 6 nm for the Gr flakes and Gr-PD flakes respectively. The lateral size of the flakes (Supp. Fig. 2b), defined by $\sqrt{LW}$ where L and W are the length and width of the flake was also investigated and showed a log-normal distribution for the Gr-PD flakes with a peak at 135 nm while the Gr flakes had a Gaussian distribution for GEth with a mean size of ~1.04 µm. For the Gr-PD, 50 flakes were counted while from the Gr flakes, 150 flakes were counted.

Sample	R_s (Ω)	C_{dl} (F)	R_{ct} (kΩ)	Z_w (Ω $(Hz)^{-0.5}$)
A-CMC	25.7	2.06E-5	101	-
A-CMC-PD	24.8	2.02E-5	176	-
A-CMC-Gr	11.6	8.47E-5	0.0462	82.2
A-CMC-PD-Gr	9.37	8.50E-5	0.0210	46.4
Carbon Foam (CF)	207	2.37E-5	41.4	-
CF-PD-Gr	57.0	2.05E-6	0.930	1670

Sup. Table 1: The equivalent circuit model of the electrochemical impedance spectroscopy data. The Warburg element set to zero for the kinetically controlled model.

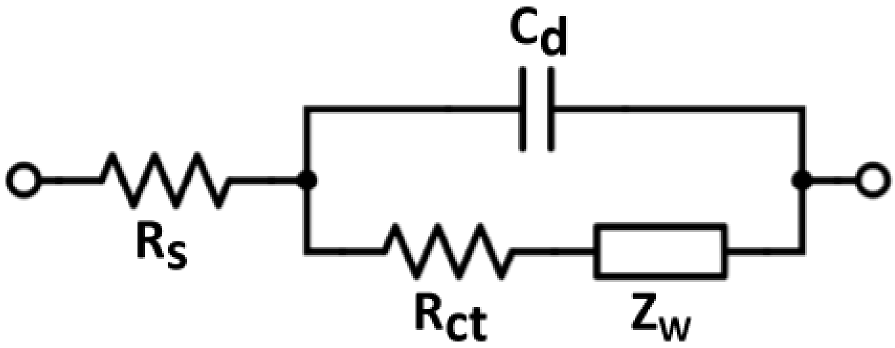

Sup. Fig. 2: The Bode equivalent circuit used describes the kinetic controlled and diffusive controlled regimes. Where R_s is the series resistance, R_{ct} is the charge transfer resistance, Cd is the double layer capacitance and Zw is the Warburg resistance.

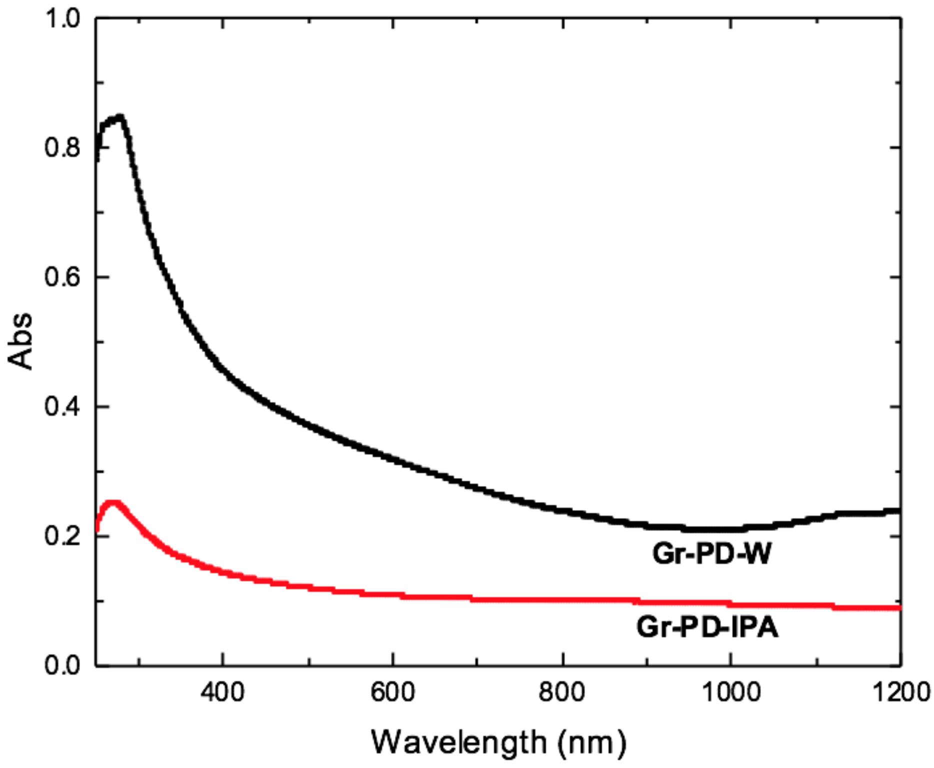

Sup. Fig. 3: Absorption spectra for Gr-PD-W and Gr-PD-IPA inks. The spectra for the graphene inks are mostly featureless due to the linear dispersion of the Dirac electrons[2,3] while the peak in the UV region is a signature of the van Hove singularity in the graphene density of states[3,4]

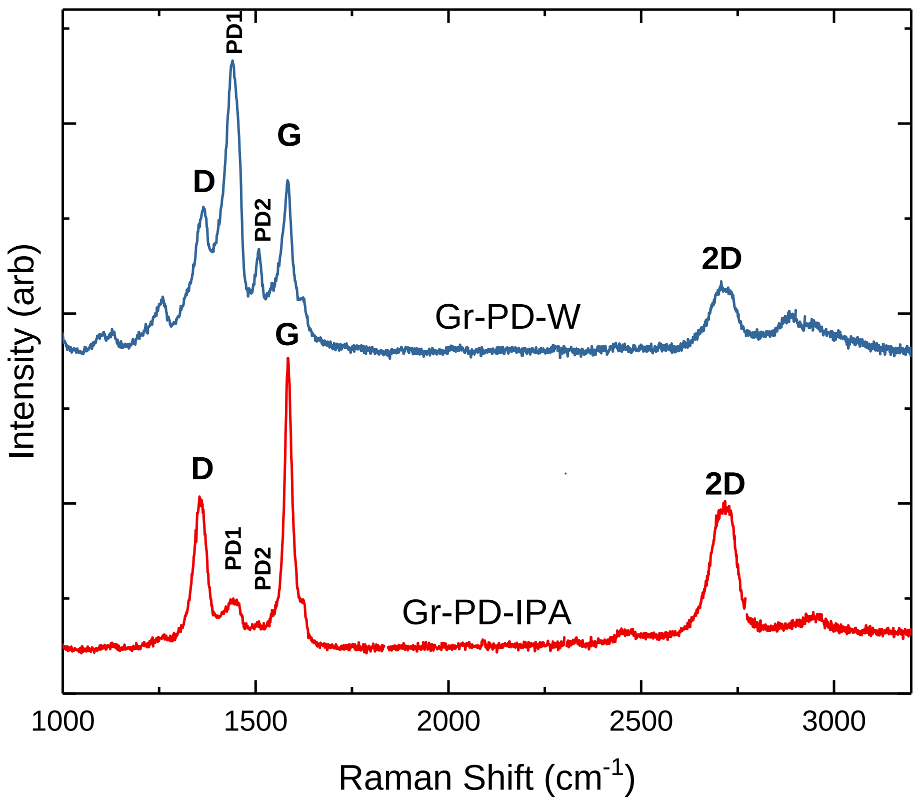

Sup. Fig. 4: Raman spectra of the Gr-PD-W flakes with the corresponding D, G and 2D peaks of the graphene located at 1359 cm^{-1}, 1582 cm^{-1} and 2713 cm^{-1} respectively. The spectra also shows two PD peaks located at 1440 cm^{-1} (PD1) and 1508 cm^{-1} (PD2). Similarly the Raman spectra of the Gr-PD-IPA ink has D, G and 2D peaks at 1356 cm^{-1}, 1584 cm^{-1} and 2713 cm^{-1} respectively in addition to PD peaks at 1442 cm^{-1} (PD1) and 1504 cm^{-1} (PD2). The peak positions of the Gr-PD-W and Gr-PD-IPA fall within the resolution of the Raman spectrometer and therefore are representative of the same material.

	SS	df	MS	F	p
Between:	61780	1	61780	158	0.000
Within:	5072	13	390		
Total:	66852	14			

Sup. Table 2: ANOVA for SS vs CF vs CF-PD-Gr MFC experiment testing the Null hypothesis that the means of the groups are the same. Comparison between the volumetric peak power outputs observed for carbon foam coated with Graphene 264.8±12.1 mW m^{-3}, n=9) compared to a graphene-free control (138.2±28.2 mW m^{-3}, n=6).

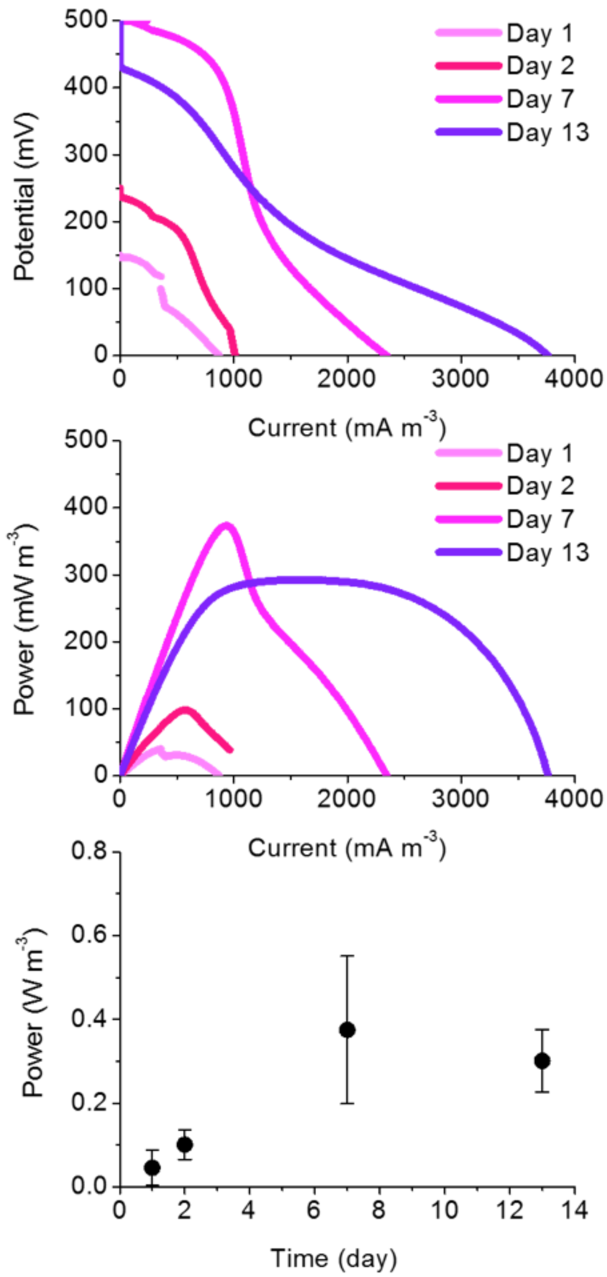

Sup. Fig. 5: Polarization and power curves of MFC devices with *R. palustris* employing both a graphene based anode and cathode over 13 days, n=3.

The associated power output of the graphene based anode (A-Gr-PD-CMC) and cathode (SS-Gr-PD) increased steadily from day 0 to day 7 due to establishment of the anodic biofilm and depletion of oxygen by respiration to generate the preferred (but not obligate) anaerobic environment for *R. palustris* until it reaches ~ 0.4 Wm^{-3} at day 7. If the value at day 13

represents a decrease, this may be due to depletion of the supplied carbon source and maturation of biofilms over the anode i.e. accumulation of dead cells affecting electron transfer. Power output fluctuations are common in MFCs employing complex biological systems[5,6], so our devices remain relatively consistent until day 13 when the experiment was completed.

References

1. Kouroupis-Agalou, K. *et al.* Fragmentation and exfoliation of 2-dimensional materials: a statistical approach. *Nanoscale* **6,** 5926–5933 (2014).

2. Mak, K. F. *et al.* Measurement of the optical conductivity of graphene. *Phys. Rev. Lett.* **101,** 196405 (2008).

3. Kravets, V. G. *et al.* Spectroscopic ellipsometry of graphene and an exciton-shifted van Hove peak in absorption. *Phys. Rev. B - Condens. Matter Mater. Phys.* **81,** (2010).

4. Chang, C. K. *et al.* Band gap engineering of chemical vapor deposited graphene by in situ BN doping. *ACS Nano* **7,** 1333–1341 (2013).

5. Inglesby, A. E., Beatty, D. a. & Fisher, A. C. Rhodopseudomonas palustris purple bacteria fed Arthrospira maxima cyanobacteria: demonstration of application in microbial fuel cells. *RSC Adv.* **2,** 4829 (2012).

6. Li, L. H., Sun, Y. M., Yuan, Z. H., Kong, X. Y. & Li, Y. Effect of temperature change on power generation of microbial fuel cell. *Environ. Technol.* **34,** 1929–1934 (2013).
